# Supplementary material for: Time-Resolved Kinetic Measurement of Microalgae Agglomeration for Screening of Polysaccharides-Based Coagulants/Flocculants
Source: Int J Environ Res Public Health. 2022 Nov 7;19(21):14610. doi: 10.3390/ijerph192114610 (PMC9657197; doi:10.3390/ijerph192114610)

Time-Resolved Kinetic Measurement of Microalgae Agglomeration for Screening of Polysaccharides-Based Coagulants/Flocculants

Supplementary Materials

Table S1 **Zeta potential and size dimensions of microalgae and polysaccharides**

Figure S1 Visible spectra of different microalgae suspensions

(a) *M. aeruginosa*; (b) *S. platensis*; (c) *S. maxima*; (d) *C. vulgaris*; (e) *I. galbana*

Figure S2 Visible spectra of different cationic polysaccharides and BEPS

Figure S3 Weber and Morris kinetic profiles of *M. aeruginosa* and *C. vulgaris* coagulation by cationic polysaccharides

(a) *M. aeruginosa*; (b) *C. vulgaris*

Figure S4 Time-resolved kinetic profiles of microalgae coagulation by AMP, PmEPS and xanthan

Table S1 Zeta potential and size dimensions of microalgae and polysaccharides

Sample	Zeta potential (mV)	Size dimensions (μm)
M. aeruginosa (OD_{680}=0.25)	- (10.5 ± 1.5)	3.33±0.24
S. platensis (OD_{680}=0.25)	- (17.1± 2.1)	29.17±1.51
S. maxima (OD_{680}=0.25)	- (14.5 ± 1.2)	33.15±2.7
C. vulgaris (OD_{680}=0.25)	- (22.7 ± 3.5)	3.378±0.15
I. galbanai (OD_{680}=0.25)	- (14.8 ± 1.2)	4.88±0.06
Agrobacterium mucopolysaccharides (AMP, 100 mg/L)	- (30.6 ± 1.8)	/
P. mucilaginosus exopolysaccharides (PmEPS, 100 mg/L)	- (29.8 ± 1.3)	/
Xanthan (100 mg/L)	- (41.3 ± 1.8)	/
Cationic hydroxyethyl cellulose (CHEC, 10 mg/L)	+ (25.6 ± 2.6)	/
Chitosan quaternary ammonium (CQA, 10 mg/L)	+ (34.6 ± 4.8)	/
Cationic guar gum (CGG, 10 mg/L)	+ (16.0 ± 1.2)	/

Figure S1 Visible spectra of different microalgae suspensions

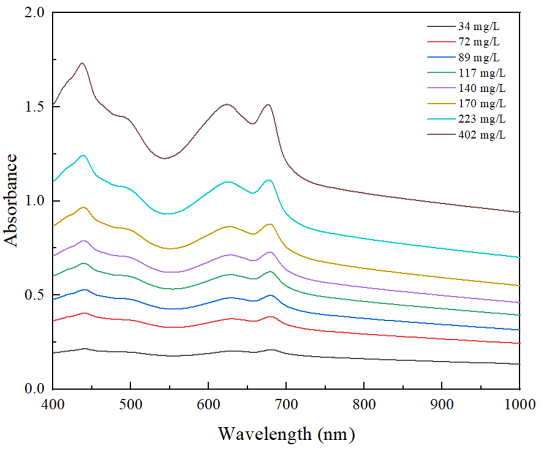

(a) *M. aeruginosa*

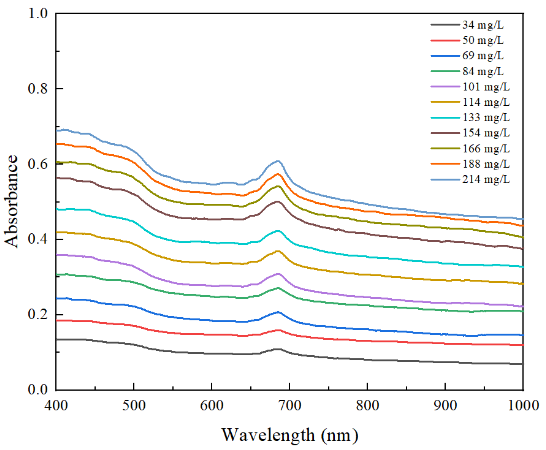

(b) *S. platensis*

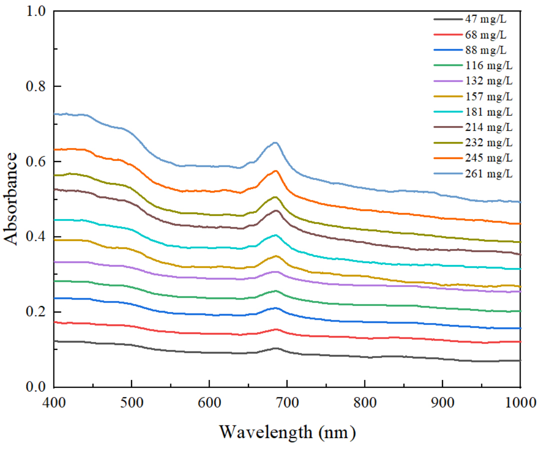

(c) *S. maxima*

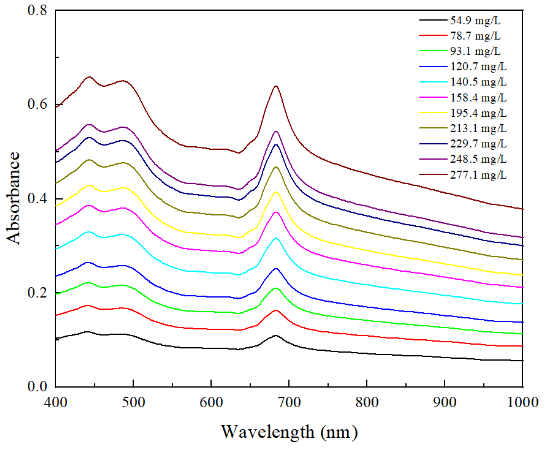

(d) *C. vulgaris*

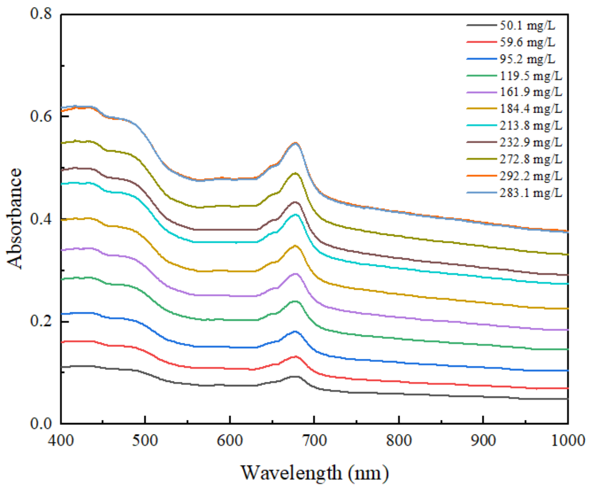

(e) *I. galbana*

Figure S2 Visible spectra of different cationic polysaccharides and BEPS

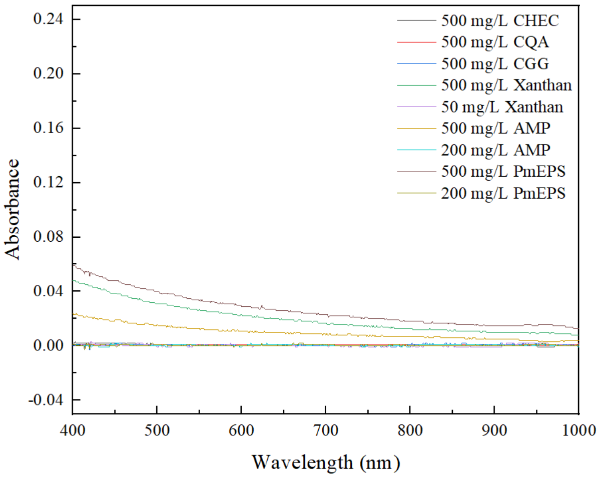

Figure S3 Weber and Morris kinetic profiles of *M. aeruginosa* and *C. vulgaris* coagulation by cationic polysaccharides

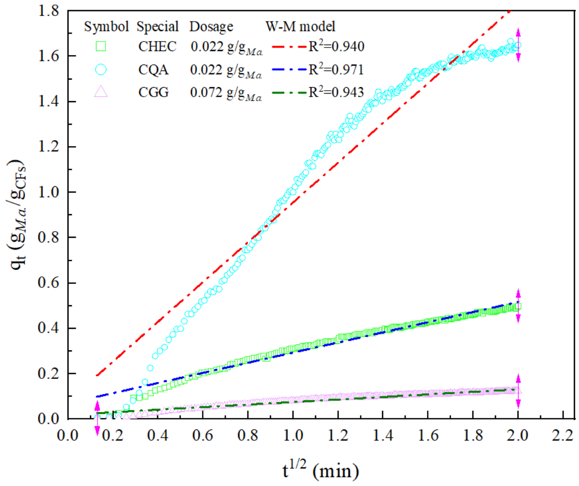

(a) *M. aeruginosa*

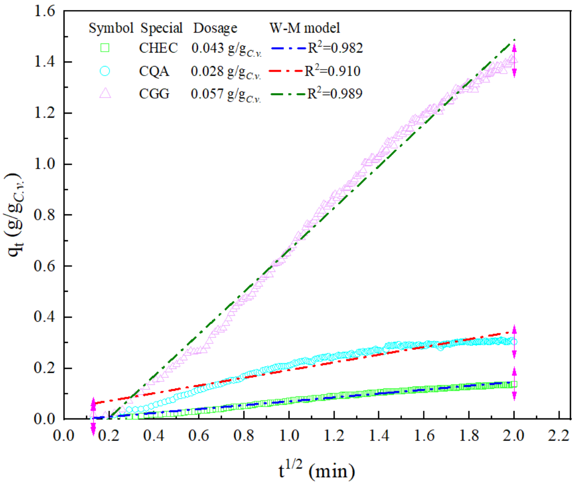

(b) *C. vulgaris*

Figure S4 Time-resolved kinetic profiles of microalgae coagulation by AMP, PmEPS and xanthan

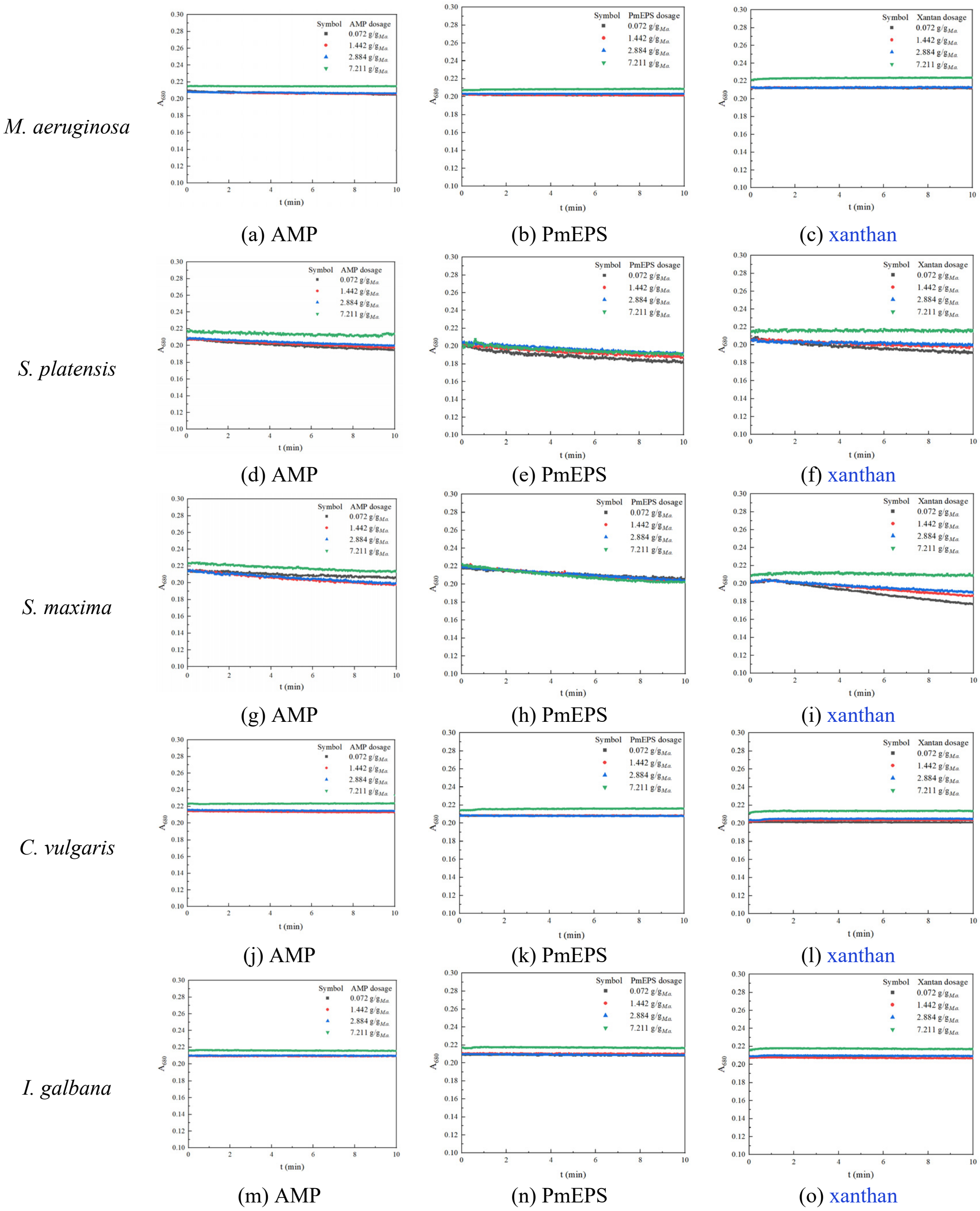

Supplement: Supplementary file 1 [file ijerph-19-14610-s001.zip › ijerph-1926280-supplementary.pdf]
